# Numerical approach for flexible body with internal boundary movement

**DOI:** 10.1038/s41598-023-32526-3

**Published:** 2023-03-31

**Authors:** Riko Ogawara, Stefan Kaczmarczyk, Yoshiaki Terumichi

**Affiliations:** 1 Division of Mechanical Engineering, Graduate School of Science and Technology, Sophia University, Tokyo, Japan; 2 Division of Technology, Department of Engineering, Faculty of Arts, Science and Technology, University of Northampton, Northamptonshire, UK

**Keywords:** Engineering, Mechanical engineering

## Abstract

In this paper, a numerical method is proposed for a flexible tether motion that spans two different environments and has large displacement and deformation. When considering the behavior of a tethered system in which the tether cable is subjected to the above conditions, variations of an internal boundary in the tether must be considered. In general, the absolute nodal coordinate formulation (ANCF), a nonlinear finite element method, is effective for the dynamic simulation of a flexible body with large displacement and deformation. However, in conventional methods, such as ANCF, the analysis accuracy decreases and the calculation cost increases when the movement of an internal boundary across different environments is considered. In this study, an efficient numerical approach that considers the variations of an internal boundary by using ANCF using variable-domain finite elements is proposed. In addition, to further improve the calculation efficiency, dimensionless variables are introduced using appropriate representative values. The accuracy of the numerical results obtained using the proposed method, which considers an internal variable boundary, is similar to that for a conventional method.

## Introduction

Tethered systems are used in various practical engineering applications^1–8^. This system is a flexible multibody system that consists of a mothership and a payload or equipment connected by a flexible tether such as a cable, rope, beam or wire. In recent years, tethered systems combining an unmanned aerial vehicle (UAV) for search, rescue, transportation, etc. are developed^9,10^ and it is believed that the scope of its use is expected to expand in the future. Such systems are used in offshore/marine exploration can across two different environments of different properties, for example, from a mother-ship in the air to an underwater payload in the sea^11^. The environmental boundary, like water/air boundary, moves relative to the tether due to the movement of the system.

**Figure 1:**
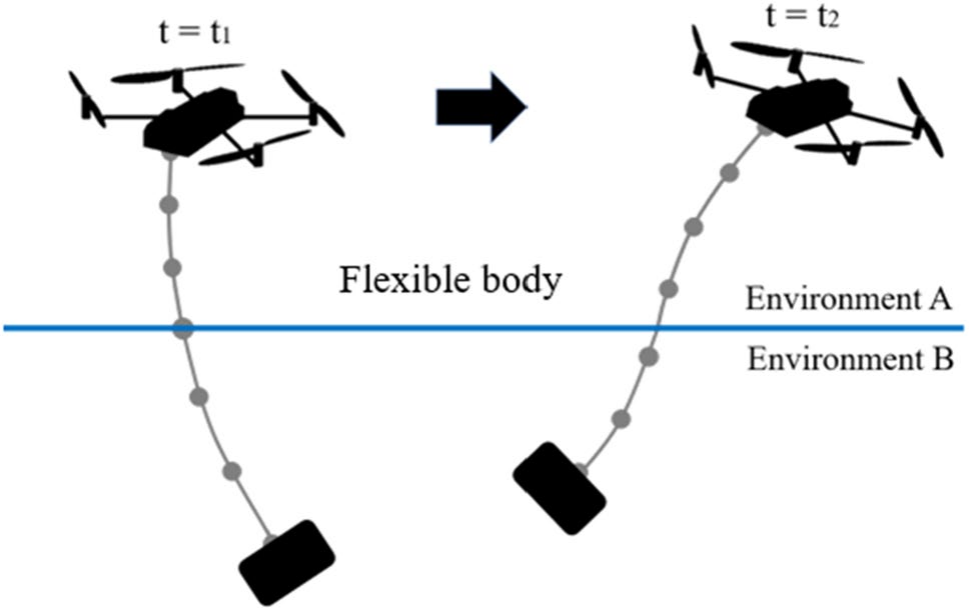
Example of model for conventional methods. Each environment in which each element exists changes as the boundary position changes. In addition, the boundary point of environment A and B is not always at a node.

Computational simulation and analysis can be used to develop models of such systems and evaluate their behavior. The absolute nodal coordinate formulation (ANCF), with a nonlinear finite element method, developed by Shabana et al.^12–17^, is widely used and developed for the dynamic simulation of flexible structures with large displacement and deformation^18–22^. However, there is no effective numerical approach for the analysis of a flexible tether with a moving environmental boundary. In the conventional methods, the equation of motion for each finite element must be re-evaluated at each time step, because the position of the internal boundary moves relative to the flexible tether, elements and nodes as shown in Fig. 1. Here, in this study, we define “the internal boundary” as the internal point of the flexible body that divides the flexible body into two parts: the part in environment A and the part in environment B (see the illustration in Fig. 1). The equation of motion depends on the environment, because the flexible tether deforms and hydrodynamic drag, buoyancy, and the added-mass effect act on the flexible tether. This re-evaluation must be conducted even if the equation of motion for a given element is the same as that in the previous time step. Therefore, a new numerical approach that can efficiently deal with such systems is required.

This study proposes a numerical approach for the analysis of a flexible body motion with an internal variable boundary. In addition, the dimensionless approach we proposed in a previous paper^23^ is applied to it. In conventional dimensionless approaches, constant representative values are generally used^24^. Compared to those conventional approaches, in our dimensionless approach, the representative values are set using the time-varying length of the flexible body. Using those time varying representative values improves calculation efficiency while maintaining accuracy since the time step is automatically changed to the appropriate value for each time-varying length of the flexible body.

The accuracy and applicability criteria of the proposed method are discussed based on a comparison of numerical results obtained using the proposed method and a conventional method.

## Modeling and the model formulation

### Analytical model

**Figure 2:**
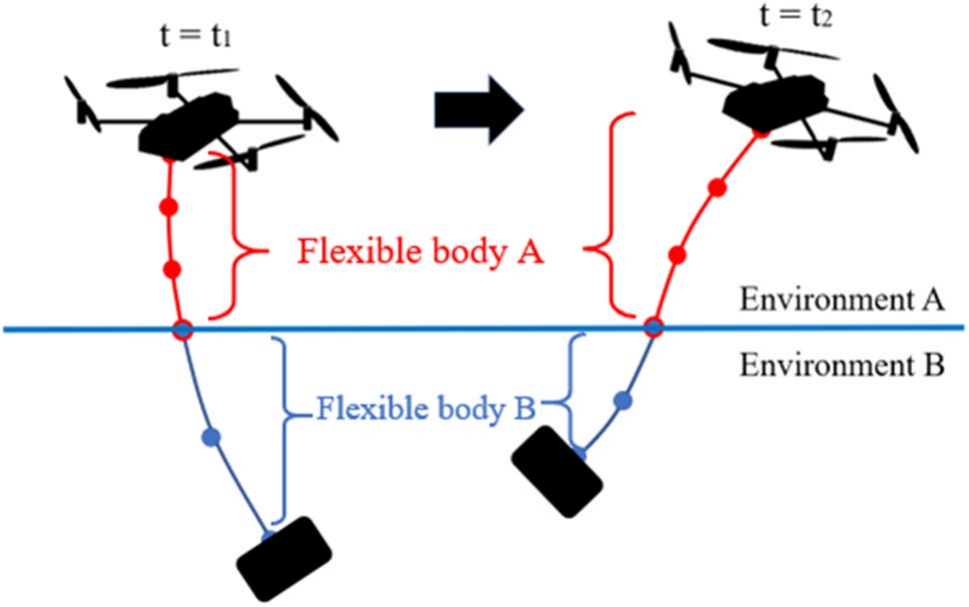
Example of model for VB-VFE-ANCF. In this model, one tether is divided into two flexible bodies, A and B, depending on the environments in which they exist. As a result, the internal boundary position is always at the connected point of flexible bodies A and B.

In the proposed method, referred to as Variable-Boundary Variable-domain Finite Element ANCF (VB-VFE-ANCF), one tether is regarded as a virtual multibody system that has two flexible bodies, namely body A above the boundary and body B below the boundary, that are combined as shown in Fig. 2. The connection point between the flexible bodies is always at the internal boundary point, because each flexible body, represented using the Variable-domain Finite Element (VFE) model^25^, changes its length relative to the movement of an internal boundary point due to deformation or displacement of the tether or movement of the mother ship. The positional relation between each node and the internal boundary, that is, above or below the boundary does not change even when the flexible tether moves and deforms. Therefore, the equation of motion for a given node is in the same form each time step. This method improves the calculation efficiency and accuracy because there is no need to search for or to approximate an internal boundary point on a flexible body.

**Figure 3:**
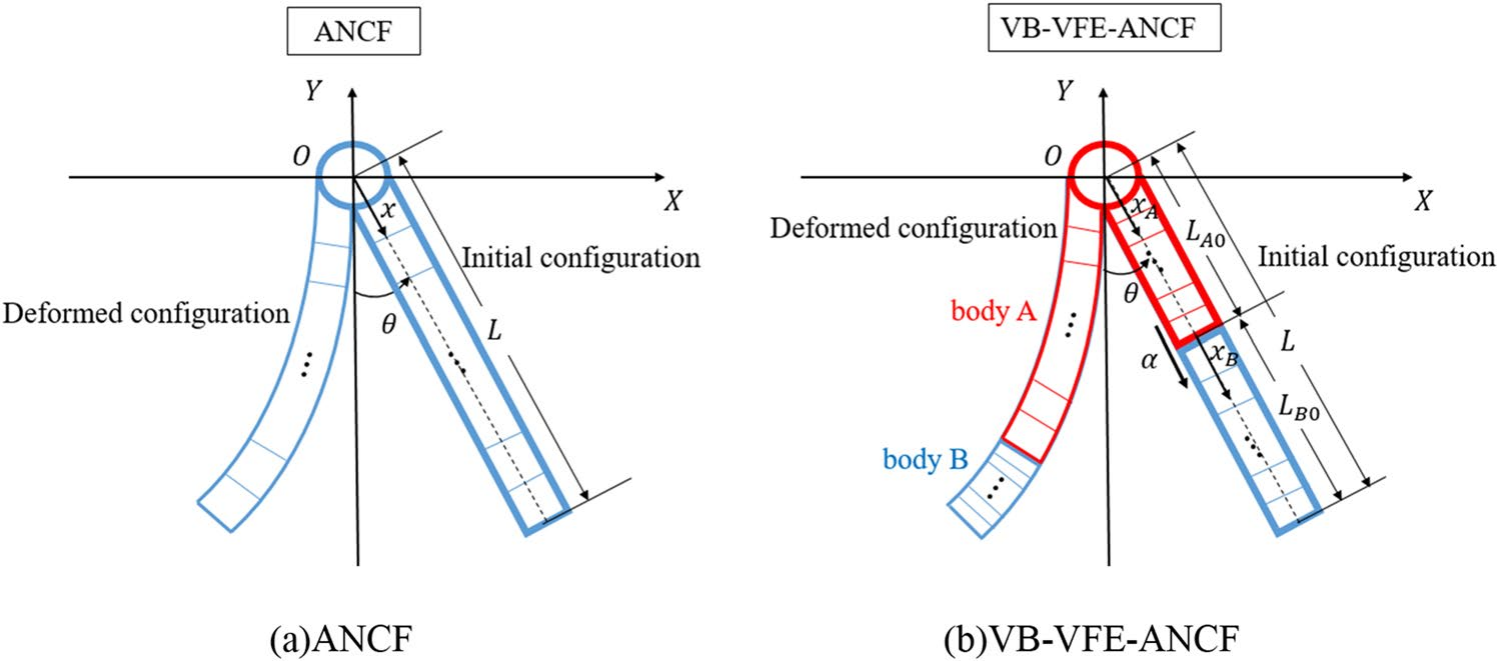
Model of a flexible pendulum. In VFE-ANCF model, each element length is same and changes evenly. On the other hands, in VB-VFE-ANCF model, the flexible pendulum was divided into two parts, and the element length of bodies A and B changes at different rates.

Figure 3 shows a flexible pendulum, which is used as a simplified analytical model in this study. The usefulness of VB-VFE-ANCF is evaluated by comparing the numerical results obtained using ANCF and VB-VFE-ANCF, which are made dimensionless by using appropriate representative values.

### Modeling and formulation of beam elements by VFE-ANCF

In this section, the modeling and formulation of VFE-ANCF, a VFE 2D model that uses ANCF, for flexible body parts that have large deformation/displacement and time-varying length are described.

Position vector ${\mathbf{r}}^{j}$ is described using the shape function $\mathbf{S}$ and the nodal coordinates ${\mathbf{e}}^{j}$ as follows

1 $$ {\mathbf{r}}^{j} = {\mathbf{Se}}^{j} $$

2 $$ {\mathbf{e}}^{j} = \left[ {\begin{array}{*{20}c} {e_{1}^{j} } & {e_{2}^{j} } & {e_{3}^{j} } & {e_{4}^{j} } & {e_{5}^{j} } & {e_{6}^{j} } & {e_{7}^{j} } & {e_{8}^{j} } \\ \end{array} } \right]^{T} $$

3 $$ {\mathbf{S}} = \left[ {\begin{array}{*{20}c} {1 - 3\xi^{2} + 2\xi^{3} } & 0 & {\xi - 2\xi^{2} + \xi^{3} } & 0 & {3\xi^{2} - 2\xi^{3} } & 0 & { - \xi^{2} + \xi^{3} } & 0 \\ 0 & {1 - 3\xi^{2} + 2\xi^{3} } & 0 & {\xi - 2\xi^{2} + \xi^{3} } & 0 & {3\xi^{2} - 2\xi^{3} } & 0 & { - \xi^{2} + \xi^{3} } \\ \end{array} } \right] $$

where $\xi =x/{l}_{e}$, $x$ is the coordinate of the point along the beam axis in the deformed configuration and ${l}_{e}$ is the length of the beam element. ${e}_{1}^{j}, {e}_{2}^{j}$ and ${e}_{5}^{j}, {e}_{6}^{j}$ represent the absolute coordinates, and ${e}_{3}^{j}, {e}_{4}^{j}$ and ${e}_{7}^{j}, {e}_{8}^{j}$ represent the absolute gradient of the nodes at the left and right ends of the element multiplied by the element length ${l}_{e}$, respectively^24,25^. These are described as follows:

4 $$ \begin{gathered} e_{3}^{j} = l_{e} \frac{{\partial r_{1} \left( {x = 0} \right)}}{\partial x}{ },\quad e_{4}^{j} = l_{e} \frac{{\partial r_{2} \left( {x = 0} \right)}}{\partial x} \hfill \\ e_{7}^{j} = l_{e} \frac{{\partial r_{1} \left( {x = l_{e} } \right)}}{\partial x}{ },\quad e_{8}^{j} = l_{e} \frac{{\partial r_{2} \left( {x = l_{e} } \right)}}{\partial x} \hfill \\ \end{gathered} $$

Here ${\dot{\mathbf{r}}}^{j}$, the time derivative of position vector ${\mathbf{r}}^{j}$, is expressed as

5 $$ {\mathbf{\dot{r}}}^{j}  = {\mathbf{S\dot{e}}}^{j}  $$

**Figure 4:**
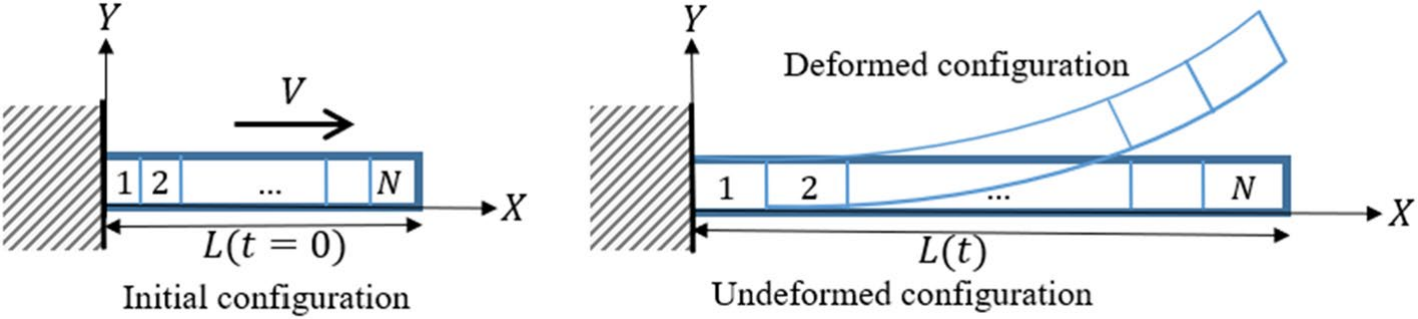
VFE model of flexible body. In the VFE-model, each element changes its length evenly.

Figure 4 shows the concept of VFE model. In the VFE method, the length of each beam element, which has a fixed number of elements $N$, changes according to the movement velocity $V$ of the flexible body with time-varying length $L(t)$. The length of beam element ${l}_{e}$ and its time derivative $\dot{{l}_{e}}$ are described as follows:

6 $$ l_{e} \left( t \right) = \frac{L\left( t \right)}{N} $$

7 $$ \dot{l}_{e} \left( t \right) = \frac{{L\left( {\dot{t}} \right)}}{N} = \frac{V}{N} $$

The length of each element changes evenly as the entire flexible body changes its length. Thus, an inertial term is generated in the equation of motion, which includes the Coriolis force due to the change in the length of the beam element.

Here, the flexible body is formulated using VFE-ANCF. The kinetic energy of an element ${T}_{e}$ can be defined using the volume $V$, density $\rho$, and cross-sectional area $A$ as follows:

8 $$ T_{e} = \frac{1}{2}\mathop \int \limits_{V}^{{}} \rho {\dot{\mathbf{r}}}^{jT} {\dot{\mathbf{r}}}^{j} dV = \frac{1}{2}\rho Al_{e} {\dot{\mathbf{e}}}^{j} \mathop \int \limits_{0}^{1} {\mathbf{S}}^{T} {\mathbf{S}}d\xi { }{\dot{\mathbf{e}}}^{j} = \frac{1}{2}\rho Al_{e} {\dot{\mathbf{e}}}^{j} {\mathbf{M}}_{{\mathbf{e}}} {\dot{\mathbf{e}}}^{j} $$

Assuming that the deformation of one element of the beam is small, the elastic energy ${U}_{e}$ *is* split into two parts, namely the elastic energy due to the axial strain ${U}_{le}$ and the elastic energy due to bending based on the curvature of the deformed beam centerline ${U}_{te}$, respectively expressed as

9 $$ \begin{gathered} U_{le} = \frac{1}{2}EAl_{e} \left\{ {\frac{1}{{l_{e}^{2} }}\left\{ {\left( {e_{5}^{j} - e_{1}^{j} } \right)^{2} + \left( {e_{6}^{j} - e_{2}^{j} } \right)^{2} } \right\}} \right. \hfill \\ \left. {\quad \quad - \frac{2}{{l_{e} }}\sqrt {\left( {e_{5}^{j} - e_{1}^{j} } \right)^{2} + \left( {e_{6}^{j} - e_{2}^{j} } \right)^{2} } + 1} \right\} \hfill \\ \end{gathered} $$

10 $$ U_{te} = \frac{1}{2}{\mathbf{e}}^{jT} \left\{ {\mathop \int \limits_{0}^{{l_{e} }} EI\left( {\frac{{\partial^{2} {\mathbf{S}}}}{{\partial x^{2} }}} \right)^{T} \left( {\frac{{\partial^{2} {\mathbf{S}}}}{{\partial x^{2} }}} \right)dx} \right\}{\mathbf{e}}^{j} $$

where $E$ is Young’s modulus, and $I$ is the second moment of inertia of the cross-sectional area. More detailed derivation of the elastic forces is explained in Ref.^26^.

The positional energy due to gravity is described as follows:

11 $$ W_{ge} = - \mathop \int \limits_{0}^{{l_{e} }} \rho A\left[ {\begin{array}{*{20}c} 0 & { - g} \\ \end{array} } \right]{\mathbf{r}}^{j} dx = - \mathop \int \limits_{0}^{{l_{e} }} \rho A\left[ {\begin{array}{*{20}c} 0 & { - g} \\ \end{array} } \right]{\mathbf{S}}dx{\mathbf{e}}^{j} $$

The kinetic energy $T$, elastic energy $U$ and gravitational energy ${W}_{g}$ of the flexible body are used in Lagrange’s equation of motion. The Lagrange’s equation of motion is expressed with the Lagrangian $L=T-U-{W}_{g}$ and the constraint reaction forces ${\mathbf{Q}}_{c}$. In VB-VFE-ANCF, as described later, ${\mathbf{Q}}_{c}$ contains the binding force generated by connecting two bodies.

12 $$ \frac{d}{dt}\left( {\frac{\partial L}{{\partial {\dot{\mathbf{e}}}}}} \right) - \left( {\frac{\partial L}{{\partial {\mathbf{e}}}}} \right) + {\mathbf{Q}}_{c} = 0 $$

Here,

13 $$ \frac{{\partial U_{le} }}{{\partial {\mathbf{e}}}} = \frac{EA}{{l_{e} }}\varepsilon_{d} {\mathbf{K}}_{le} {\mathbf{e}} = \frac{EA}{{l_{e} }}\varepsilon_{d} \left[ {\begin{array}{*{20}c} 1 & {} & {} & {} & {} & {} & {} & {} \\ 0 & 1 & {} & {} & {} & {} & {sym.} & {} \\ 0 & 0 & 0 & {} & {} & {} & {} & {} \\ 0 & 0 & 0 & 0 & {} & {} & {} & {} \\ { - 1} & 0 & 0 & 0 & 1 & {} & {} & {} \\ 0 & { - 1} & 0 & 0 & 0 & 1 & {} & {} \\ {\begin{array}{*{20}c} 0 \\ 0 \\ \end{array} } & {\begin{array}{*{20}c} 0 \\ 0 \\ \end{array} } & {\begin{array}{*{20}c} 0 \\ 0 \\ \end{array} } & {\begin{array}{*{20}c} 0 \\ 0 \\ \end{array} } & {\begin{array}{*{20}c} 0 \\ 0 \\ \end{array} } & {\begin{array}{*{20}c} 0 \\ 0 \\ \end{array} } & {\begin{array}{*{20}c} 0 \\ 0 \\ \end{array} } & {\begin{array}{*{20}c} {} \\ 0 \\ \end{array} } \\ \end{array} } \right]{\mathbf{e}} $$

14 $$ \frac{{\partial U_{te} }}{{\partial {\mathbf{e}}}} = \frac{EI}{{l_{e}^{3} }}{\mathbf{K}}_{te} {\mathbf{e}} = \frac{EI}{{l^{3} }}\left[ {\begin{array}{*{20}c} {12} & {} & {} & {} & {} & {} & {} & {} \\ 0 & {12} & {} & {} & {} & {} & {sym.} & {} \\ 6 & 0 & 4 & {} & {} & {} & {} & {} \\ 0 & 6 & 0 & 4 & {} & {} & {} & {} \\ {\begin{array}{*{20}c} { - 12} \\ 0 \\ \end{array} } & {\begin{array}{*{20}c} 0 \\ { - 12} \\ \end{array} } & {\begin{array}{*{20}c} { - 6} \\ 0 \\ \end{array} } & {\begin{array}{*{20}c} 0 \\ { - 6} \\ \end{array} } & {\begin{array}{*{20}c} {12} \\ 0 \\ \end{array} } & {\begin{array}{*{20}c} {} \\ {12} \\ \end{array} } & {\begin{array}{*{20}c} {} \\ {} \\ \end{array} } & {} \\ 6 & 0 & 2 & 0 & { - 6} & 0 & 4 & {} \\ 0 & 6 & 0 & 2 & 0 & { - 6} & 0 & 4 \\ \end{array} } \right]{\mathbf{e}} $$

15 $$ \frac{{\partial W_{ge} }}{{\partial {\mathbf{e}}}} = - \rho Al_{e} g{\mathbf{C}}_{ge} = - \rho Al_{e} g\left[ {\begin{array}{*{20}c} 0 \\ {1/2} \\ 0 \\ {1/12} \\ 0 \\ {1/2} \\ 0 \\ { - 1/12} \\ \end{array} } \right] $$

From the above, the equation of motion for a beam element is expressed as follows:

16 $$ {\mathbf{M}}_{e} {\mathbf{\ddot{e}}} + \frac{{\dot{l}_{e} }}{{l_{e} }}{\mathbf{M}}_{e} {\mathbf{\dot{e}}} + \frac{E}{{\rho l_{e}^{2} }}\varepsilon _{d} {\mathbf{K}}_{{le}} {\mathbf{e}} + \frac{{EI}}{{\rho Al_{e}^{4} }}{\mathbf{K}}_{{te}} {\mathbf{e}} + g{\mathbf{C}}_{{ge}}  + {\mathbf{Q}}_{c}  = 0 $$

where ${\mathbf{K}}_{le}$ ${\mathbf{K}}_{te}$, and ${\mathbf{C}}_{ge}$ are constants and ${\varepsilon }_{d}$ is defined as

17 $$ \varepsilon_{d} = \frac{{\sqrt {\left( {e_{5} - e_{1} } \right)^{2} + \left( {e_{6} - e_{2} } \right)^{2} } - l_{e} }}{{\sqrt {\left( {e_{5} - e_{1} } \right)^{2} + \left( {e_{6} - e_{2} } \right)^{2} } }} $$

Here, the equation of motion of each element are synthesized in augmented form as follows;

18 $$ \left[ {\begin{array}{*{20}c}    {\mathbf{M}} & {{\mathbf{C}}_{q}^{T} }  \\    {{\mathbf{C}}_{q} } & 0  \\   \end{array} } \right]\left[ {\begin{array}{*{20}c}    {{\mathbf{\ddot{q}}}}  \\    {\mathbf{\lambda }}  \\   \end{array} } \right] = \left[ {\begin{array}{*{20}c}    {\mathbf{Q}}  \\    {\mathbf{\gamma }}  \\   \end{array} } \right] $$

the constraint force in Eq. (16) is derived from ${\mathbf{Q}}_{c} = {{\mathbf{C}}_{q}^{T} }{\mathbf{\lambda }}$, where $\mathbf{C}$ is the constraints equations, ${\mathbf{C}}_{q}$ is the Jacobian matrix of it, $\mathbf{M}$ is the mass matrix of the system, $\mathbf{q}$ is the vector of the system generalized coordinates, $\mathbf{Q}$ is the vector of external forces and ${\mathbf{C}}_{q} {\mathbf{\ddot{q}}} = {{\varvec{\upgamma}}}$^15^.

In Eq. (16), ${\mathbf{M}}_{e}$ and ${\mathbf{C}}_{ge}$ are made constants by using the nodal coordinates defined by Eq. (4), reducing the calculation cost. The second term on the left-hand side of Eq. (16) represents the inertial force caused by the change in length.

### Proposed method for flexible body motion with variable boundary

In this section, the dimensionless equation of motion and the method for determining the connected position of bodies A and B (see the flexible pendulum model in Fig. 3), that is, the length of bodies according to the change in the internal boundary position in the VB-VFE-ANCF formulation, are described. In this study, as a simple example for basic consideration, the equilibrium length, the unstretched length when no external forces are presented, of the entire length of a flexible tether $L={L}_{A}\left(t\right)+ {L}_{B}(t)$ is constant and the internal boundary point moves with constant velocity $\alpha \;{\text{m}}/{\text{s}}$. The case that a system that a drone and a machine is connected via flexible tether like Fig. 1 descends at constant velocity is one of the examples.

Therefore, the length of body *i*, ${L}_{i}\left(t\right)$, is defined as follows:

19 $$ L_{i} \left( t \right) = L_{i0} + \alpha_{i} t $$

where $i=A, B$, ${\alpha }_{A}=\alpha , {\alpha }_{B}=-\alpha$, and ${L}_{i0}$ represents the initial length of body *i*.

The dimensionless variables are defined as follows.

20 $$ {\mathbf{e}}_{A} = L_{A} \left( t \right){\mathbf{e}}_{A}^{*} , {\mathbf{e}}_{B} = L_{B} \left( t \right){\mathbf{e}}_{B}^{*} , x_{A} = L_{A} \left( t \right)x_{A}^{*} ,x_{B} = L_{B} \left( t \right)x_{B}^{*} , t = T_{R} t^{*} $$

Here, the time-varying length of each body ${L}_{A}\left(t\right)$ and ${L}_{B}(t)$, respectively, is used as the representative length ${L}_{R}$, and the representative time ${T}_{R}$ is described as ${T}_{R}={L}_{s}(t)\sqrt{\rho /E}$, where ${L}_{s}(t)$ is the shorter length of the body lengths ${L}_{A}(t)$ and ${L}_{B}(t)$, respectively. In addition, ${\mathbf{e}}_{A}$ and ${\mathbf{e}}_{B}$ are the nodal coordinate of body A and B, respectively.

Thus, the dimensionless equation of motion is derived by applying the above body length in Eq. (19) and dimensionless variables to Eq. (16) (details are in Appendix 1). In addition, a dimensionless function ${\mu }_{s}^{*}={L}_{s}\left({t}^{*}\right)/{L}_{s0}=1/(1-{\alpha }_{s}^{*}{t}^{*})$ is introduced as an indicator expressing the amount of internal boundary movement with respect to the initial length.

21 $$ \begin{gathered}   \mathop \int \limits_{0}^{1} {\mathbf{S}}^{T} {\mathbf{S}}d\xi {\ddot{\mathbf{e}}}_{i} ^{{\text{*}}}  + \left( {3\frac{{\mu _{s}^{{\text{*}}} }}{{\lambda _{{is}}^{{\text{*}}} }}\alpha _{i}^{{\text{*}}}  - 2\mu _{s}^{{\text{*}}} \alpha _{s}^{{\text{*}}} } \right)\mathop \int \limits_{0}^{1} {\mathbf{S}}^{T} {\mathbf{S}}d\xi {\dot{\mathbf{e}}}_{i} ^{{\text{*}}}  + \frac{{\alpha _{i}^{{{\text{*}}2}} \mu _{s}^{{{\text{*}}2}} }}{{\lambda _{{is}}^{{{\text{*}}2}} }}\mathop \int \limits_{0}^{1} {\mathbf{S}}^{T} {\mathbf{S}}d\xi {\mathbf{e}}_{i}^{*}  \hfill \\   \quad  + \frac{{\mu _{s}^{{{\text{*}}2}} }}{{\lambda _{{is}}^{{{\text{*}}2}} l_{{ei}}^{{{\text{*}}2}} }}\varepsilon _{{di}}^{{\text{*}}} {\mathbf{K}}_{{lei}} {\mathbf{e}}_{i}^{*}  + \frac{{d_{i}^{{{\text{*}}2}} \mu _{s}^{{{\text{*}}2}} }}{{16\lambda _{{is}}^{{{\text{*}}2}} l_{{ei}}^{{{\text{*}}4}} }}{\mathbf{K}}_{{tei}} {\mathbf{e}}_{i}^{*}  + \frac{{\mu _{s}^{{{\text{*}}2}} g_{i}^{{\text{*}}} }}{{\lambda _{{is}}^{{{\text{*}}2}} }}{\mathbf{C}}_{{ge}}  + {\mathbf{Q}}_{{ci}}^{{\prime {\text{*}}}}  = 0 \hfill \\  \end{gathered}  $$

The superscript asterisk indicates a dimensionless variable. The dimensionless parameters are defined as follows:

22 $$ \alpha_{i}^{*} = \alpha_{i} \sqrt {\frac{\rho }{E}} ,{ }\alpha_{s}^{*} = \alpha_{s} \sqrt {\frac{\rho }{E}} ,{ }\lambda_{is}^{*} = \frac{{L_{i} \left( t \right)}}{{L_{s} \left( t \right)}}{ },{ }g_{i}^{*} = \frac{{\rho L_{i} \left( t \right)g}}{E}{ },{ }d_{i}^{*} = \frac{D}{{L_{i} \left( t \right)}}, { }l_{ei}^{*} = \frac{1}{{N_{i} }}, $$

where $D$ is the cross-sectional diameter and ${\alpha }_{i}^{*}$ is a dimensionless parameter that represents the relative internal boundary movement speed to the propagation speed of longitudinal waves. The constraints equation of bodies A and B are given as follows:

23 $$ {\mathbf{C}} = \left[ {\begin{array}{*{20}c} {e_{A}^{{*(4N_{A} + 1)}} - e_{B}^{*\left( 1 \right)} } \\ {e_{A}^{{*\left( {4N_{A} + 2} \right)}} - e_{B}^{*\left( 2 \right)} } \\ {e_{A}^{{*(4N_{A} + 3)}} - e_{B}^{*\left( 3 \right)} } \\ {e_{A}^{{*\left( {4N_{A} + 4} \right)}} - e_{B}^{*\left( 4 \right)} } \\ \end{array} } \right] = 0 $$

where ${N}_{A}$ and ${N}_{B}$ are the numbers of elements of the respective body.

By making the equation of motion dimensionless, the physical factors that govern complex behavior are normalized and movements can be more appropriately evaluated. In addition, as shown in a previous study^23^, by setting the representative length to the length of each body that changes in time, flexible body is converted to the dimensionless system consisting of two bodies with constant length. Furthermore, by setting the representative time using the time-varying length of the shorter body, the time step is set according to the higher frequency of bodies A and B at each time step. Here, in this study, change in the representative time ${T}_{R}$ means change in the dimensional time step $\Delta t$, defined in Eq. (20), since the dimensionless time step $\Delta {t}^{*}$ is constant. In other words, the dimensional time step automatically changes to the value according to the shorter body length with the higher frequency for each calculation time step.

As a result, it is possible to perform numerical analysis while maintaining accuracy regardless of the change in length.

## Numerical results and discussion

In this section, a numerical analysis of a flexible body with a variable boundary is performed using the proposed method (VB-VFE-ANCF). The numerical results obtained using VB-VFE-ANCF are compared with those obtained using ANCF, which is generally considered to be effective for dynamic flexible body simulations without a variable boundary. Here, for VB-VFE-ANCF, the environments in which the two bodies exist are the same, so the results should be in good agreement with those of ANCF. Moreover, the analysis accuracy and criteria for applicability of the proposed method are discussed based on the difference in the numerical results between the two methods. (The comparison of the computational time is shown in Appendix 2).

### Condition of numerical analysis model

In this paper, a numerical analysis of the planar motion of three models, a free falling of a very flexible beam model under gravity (model 0), a flexible pendulum model with length $L$ under gravity, as shown in Fig. 3 (model I) and a flexible beam model with length $L$ under zero gravity (model II) are performed.

A free falling of very flexible beam model (model 0)^27–29^:

The beam can rotate with the upper end being pivoted at the origin $O$ of the absolute coordinate system $XY$. The beam has a length of 1.2 m, a diameter of 0.05 m, a density of 5540 $\mathrm{kg}/{\mathrm{m}}^{3}$ and a modulus of elasticity of $0.7\times {10}^{6}$ Pa. In the initial state, the flexible beam is horizontal and has zero velocity.

A flexible pendulum model (model I): The pendulum can rotate with the upper end being pivoted at the origin $O$ of the absolute coordinate system $XY$. The pendulum is made of fluorocarbon, and it has a length of $L$ m, a diameter of 0.001 m, a density of 1780 $\mathrm{kg}/{\mathrm{m}}^{3}$ and a modulus of elasticity of $1.3\times {10}^{9}$ Pa^30^. In the initial state, the flexible pendulum is placed at an angle of 30° with respect to the vertical direction and has zero velocity.

A flexible beam model (model II): The right and left end of the flexible beam are constrained to rotate freely at the origin $O$ of the absolute coordinate system $XY$ and $(L, 0)$. The beam has a length of $L$ m, a diameter of 0.001 m, a density of 5540 $\mathrm{kg}/{\mathrm{m}}^{3}$ and a modulus of elasticity of $0.7\times {10}^{6}$ Pa. In the initial state, the flexible beam is given a deformation of sine curve shape with an amplitude of 0.1 m in a vertical direction and has zero velocity.

In VB-VFE-ANCF, flexible bodies A and B have initial lengths ${L}_{A0}$, and ${L}_{B0}$, respectively, and the length of each body changes with internal boundary movement speed $\alpha$. Numerical calculations were conducted using the fourth-order Runge–Kutta method and the force exerted by the environment are neglected in those models. In addition, the dimensionless equation of motion derived in the previous chapter is used in models I and II. However, in model 0, the dimensional equation of motion is used to compare under the same conditions as Refs.^27–29^.

### The effect of the dimensionless approach

**Figure 5:**
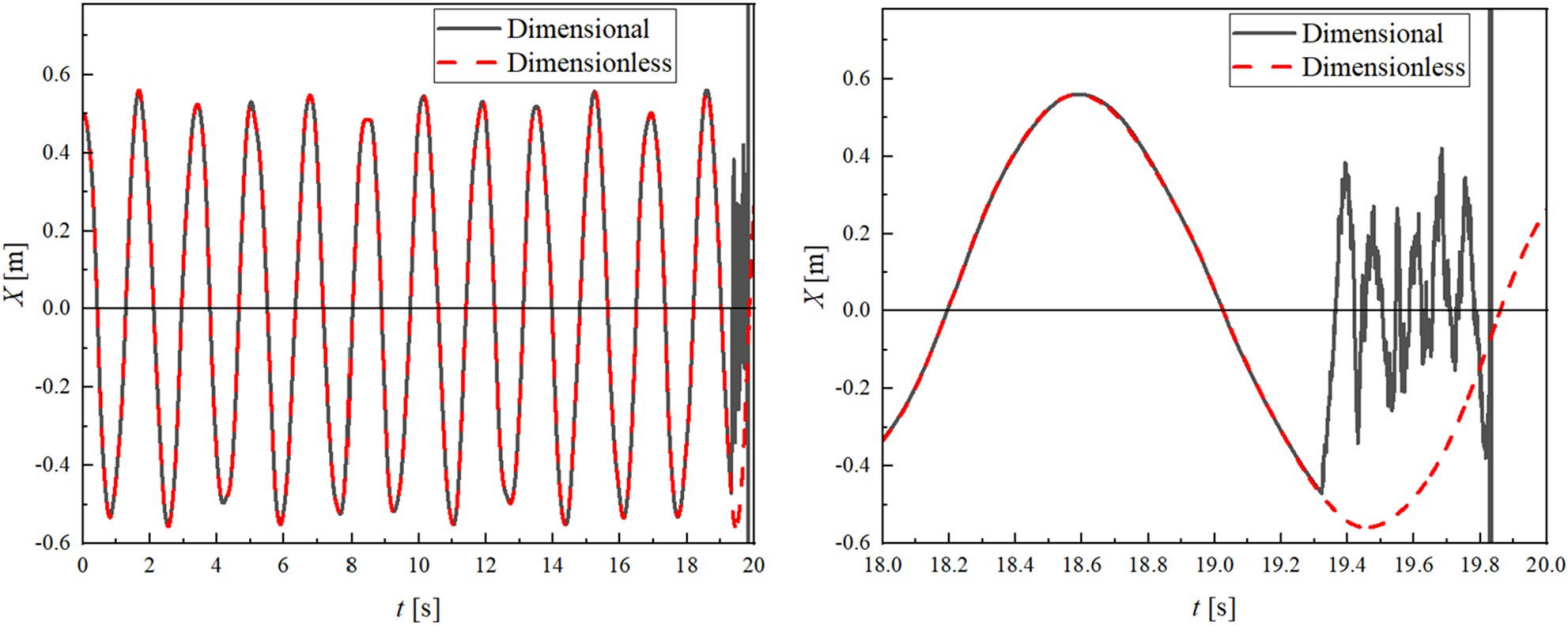
Time history of end point coordinate in X direction (left) and enlarged view (right). The dimensionless case maintains high accuracy compared to the dimensional case when a body length becomes shorter and the frequency becomes higher.

Here, the aforementioned effect of the dimensionless approach is shown in Fig. 5. This figure shows the result of the flexible pendulum model (model I) where ${L}_{A0}={L}_{B0}=0.5$ m, $\alpha =-0.02$ m/s and ${N}_{A}={N}_{B}=10$.

The diagrams shown in this figure demonstrate the time history of horizontal displacement of the end point of the pendulum when the initial time steps $\Delta t$ are set to be equally, $\Delta t=1.0\times {10}^{-5}$, and the dimensional and dimensionless equations of motion are used. It can be seen that accuracy drops significantly around 19.3 s in the dimensional case, whereas that in the dimensionless case is maintained.

In addition, for the second and third terms on the left-hand side of the equation of the motion, Eq. (21), each parameter is derived from the following equation.

24 $$ \frac{{\alpha_{i}^{*} }}{{\lambda_{is}^{*} }}\mu_{s}^{*} = \frac{1}{{L_{R} }}\frac{{dL_{R} }}{{dt^{*} }}, \alpha_{s}^{*} \mu_{s}^{*} = \frac{1}{{T_{R} }}\frac{{dT_{R} }}{{dt^{*} }} $$

The apparent inertial force occurs because the representative values of length and time change with respect to dimensionless time. The change in the representative values of space and time means that the movement speed of the internal boundary position $\alpha \ne 0$. That is, the inertial forces are generated because of the internal boundary movement.

Therefore, in the next section, the relationship between the parameters in the inertial force and the difference between the numerical results for ANCF and VB-VFE-ANCF is shown.

### Comparison of analysis results obtained using ANCF and VB-VFE-ANCF

In this section, the numerical results obtained using ANCF and VB-VFE-ANCF are compared. The effect on the analysis results of expressing the movement of the internal boundary position by the change in the length of each body is shown.

**Figure 6:**
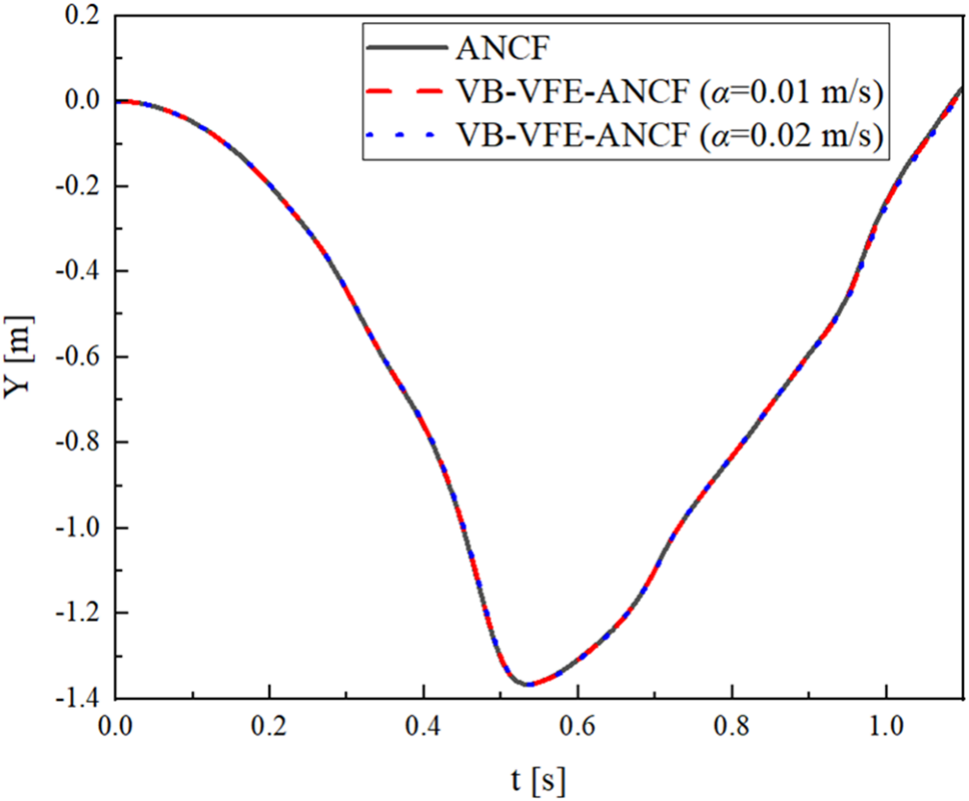
Comparison of the proposed method and conventional method ANCF using free-falling model (model 0) which is widely-used as a flexible beam problem. These results are in good agreement with the results shown in Refs. ^27–29^.

First, the comparison of the conventional method, ANCF, and the proposed method, VB-VFE-ANCF using free-falling model (model 0) is shown in Fig. 6. This figure shows that the proposed method can accurately express the motion of a very flexible beam model as well as conventional method^27–29^.

**Figure 7:**
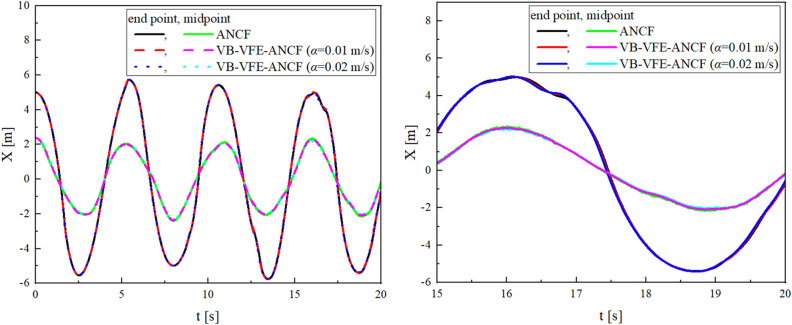
Comparison of X coordinate values of flexible pendulum obtained using ANCF and VB-VFE-ANCF (left), and enlarged view (right). These results are in good agreement. This shows that it is possible to obtain the numerical analysis results with the same accuracy as ANCF by using VB-VFE-ANCF.

**Figure 8:**
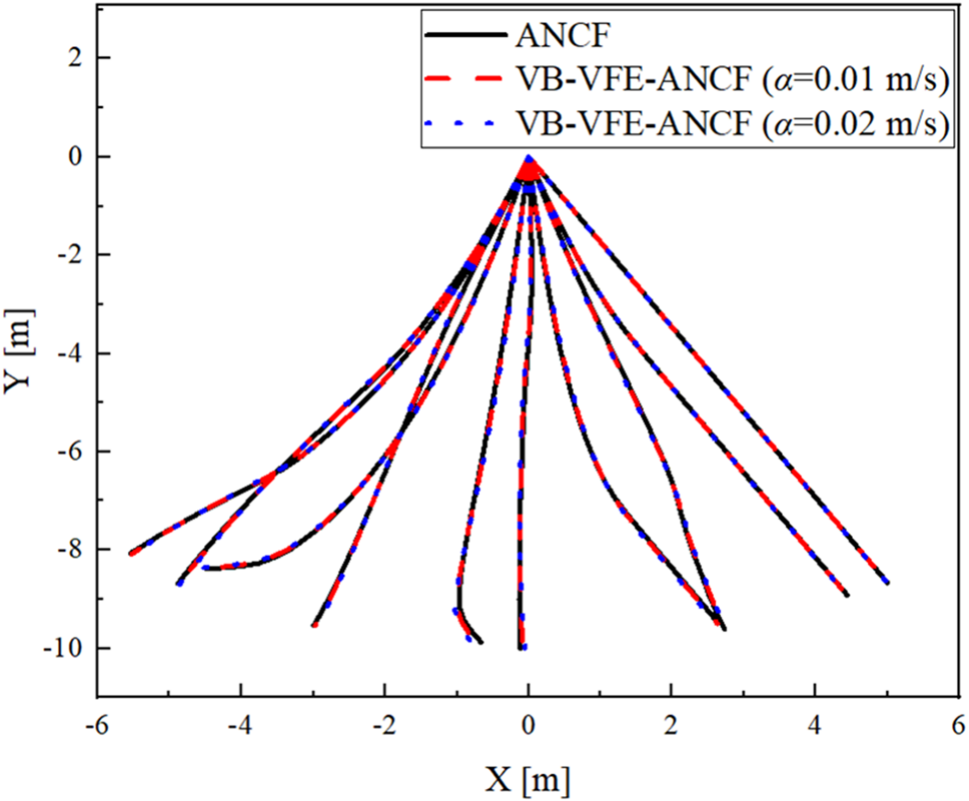
Comparison of the flexible pendulum shape obtained using ANCF and VB-VFE-ANCF for 5 s every 0.5 s.

Figures 7 and 8 show a comparison of the end point and midpoint displacement and the shape obtained using flexible pendulum model (model I), where $L=10.0$ m and $N=$ 40 for ANCF and ${L}_{A0}={L}_{B0}=5.0$ m, ${N}_{\mathrm{A}}={N}_{B}=20$ and internal boundary movement speed $\alpha =0.1, 0.2$ m/s for VB-VFE-ANCF. The initial time steps $\Delta t$ are set to $\Delta t=1.0\times {10}^{-5}$ in all these cases.

**Figure 9:**
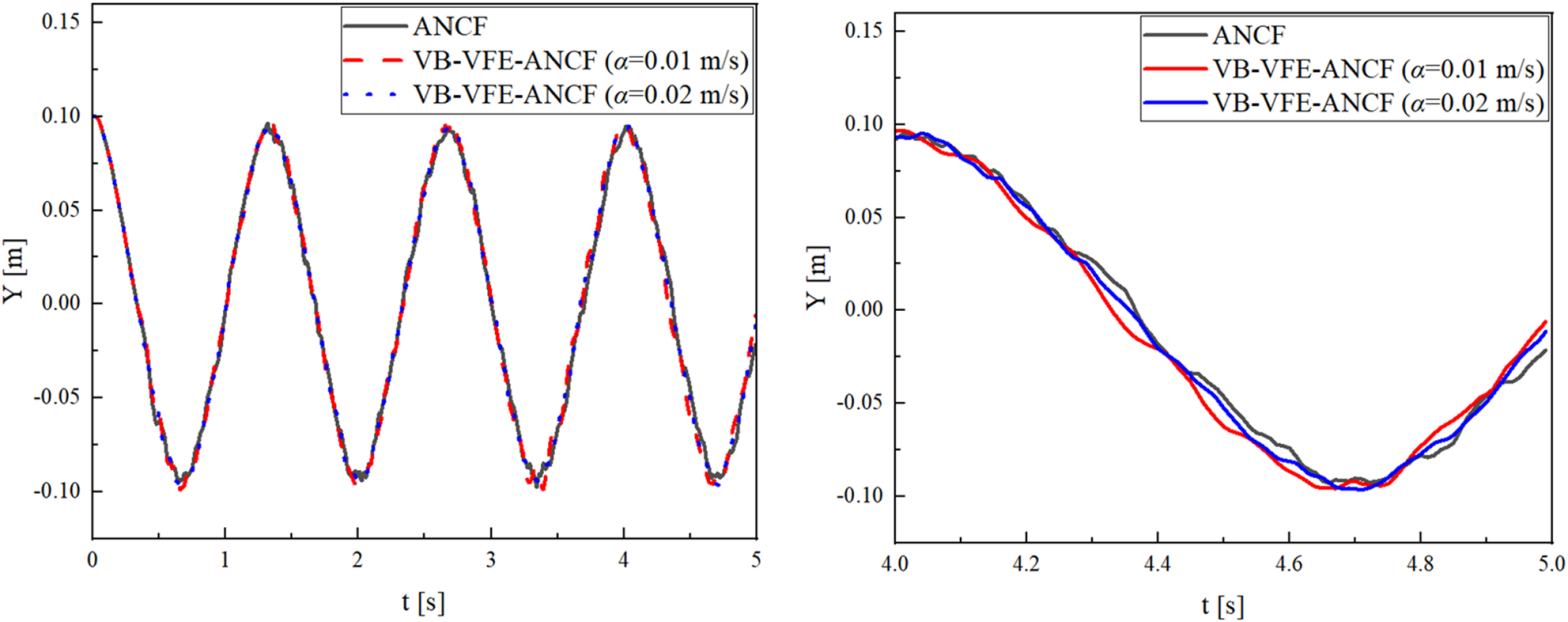
Comparison of Y coordinate values of flexible beam obtained using ANCF and VB-VFE-ANCF (left), and enlarged view (right). These results show that VB-VFE-ANCF is effective for the very flexible beam model with large deformation.

**Figure 10:**
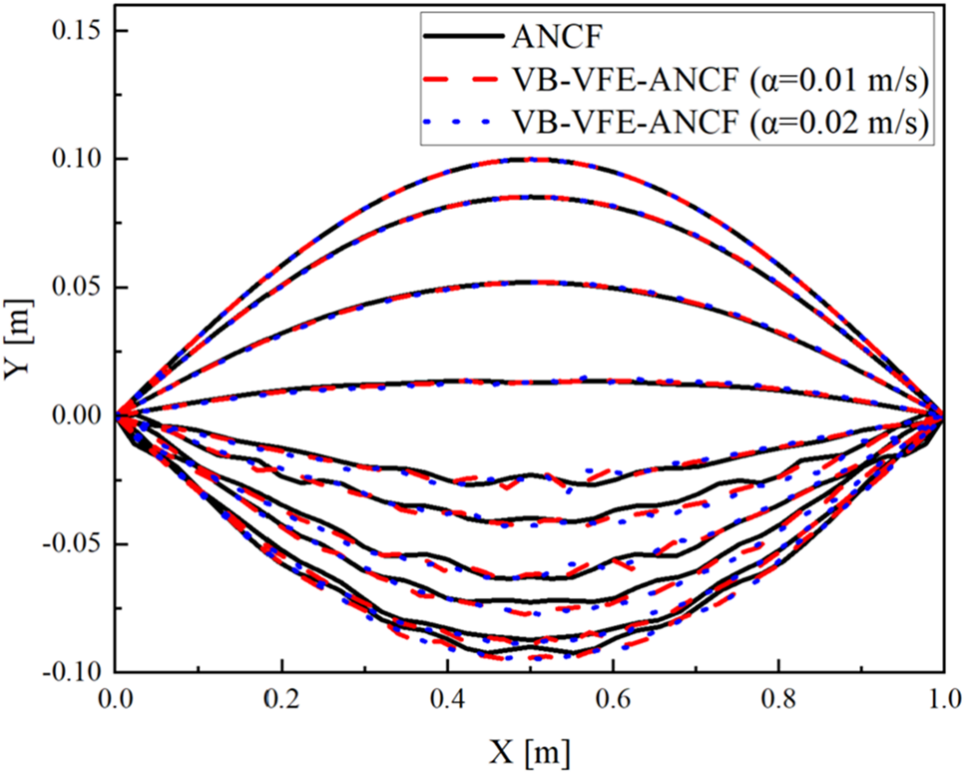
Comparison of the flexible beam shape obtained using ANCF and VB-VFE-ANCF for 1 s every 0.1 s.

Figures 9 and 10 shows a comparison of the midpoint displacement and the shape obtained using beam model (model II), where $L=1.0$ m and $N=$ 40 for ANCF and ${L}_{A0}={L}_{B0}=0.5$ m, ${N}_{\mathrm{A}}={N}_{B}=20$ and internal boundary movement speed $\alpha =0.01, 0.02$ m/s for VB-VFE-ANCF. The time step $\Delta t$ is set to $\Delta t=1.0\times {10}^{-6}$ in all these cases.

As shown, there are slight differences in the results of ANCF and VB-VFE-ANCF. This difference depends on the internal boundary movement speed $\alpha$ because of the inertial force generated by the internal boundary movement described in the second and third terms on the left-hand side of the equation of motion, Eq. (21).

### Evaluation of accuracy and applicability of proposed method

Here, the usefulness of the proposed method is evaluated and the scope of application is examined using an analytical model that moves as a pendulum due to gravity (model I). The difference ${\varepsilon }^{*}$ between the numerical results of ANCF and VB-VFE-ANCF, expressed by the following equation, is used as an indicator of accuracy.

25 $$ \varepsilon^{*} = \frac{{\sqrt {\left( {X - X_{C} } \right)^{2} + \left( {Y - Y_{C} } \right)^{2} } }}{L} \times 100 $$

where ${X}_{C}$ and ${Y}_{C}$ are the displacements obtained using ANCF, and $X$ and $Y$ are those obtained using VB-VFE-ANCF, denoted by Eq. (21). Here, the number of elements and the initial time steps are set to $N=40$ for ANCF, ${N}_{\mathrm{A}}={N}_{B}=20$ for VB-VFE-ANCF and $\Delta t=1.0\times {10}^{-5}$ in all cases below.

**Figure 11:**
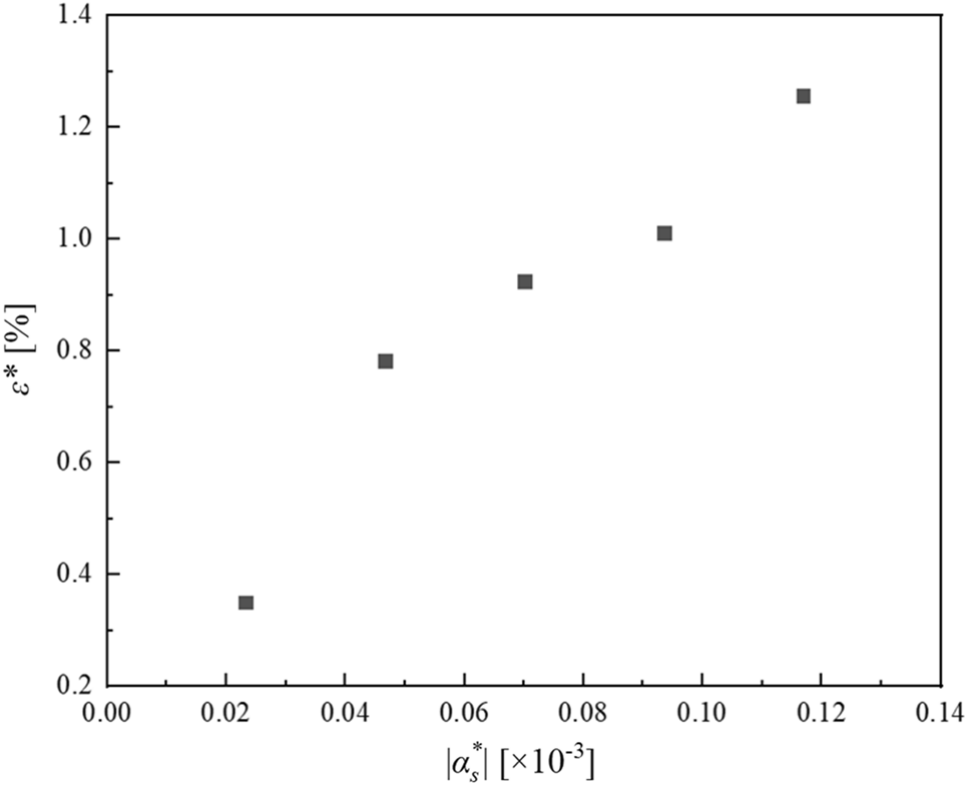
Relationship between ${\alpha }_{s}^{*}$ and ${\varepsilon }^{*}$.

**Figure 12:**
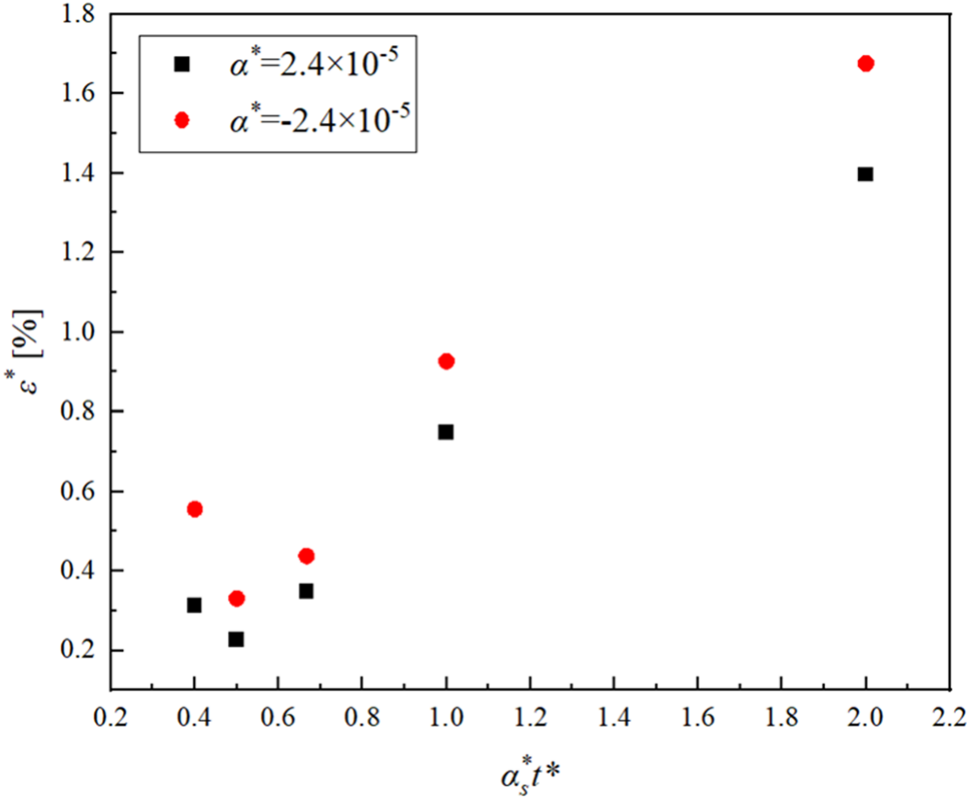
Relationship between ${\alpha }_{s}^{*}{t}^{*}$ and ${\varepsilon }^{*}$.

Figures 11 and 12 show the change of ${\varepsilon }^{*}$ in the cases of ${\alpha }_{s}^{*}{t}^{*}$ is fixed and ${\alpha }_{s}^{*}$ is varied, or ${\alpha }_{s}^{*}$ is fixed and ${\alpha }_{s}^{*}{t}^{*}$ is varied respectively. In order to clarify the effect of the inertial force generated by the internal boundary movement on the numerical analysis accuracy, the parameters in the term of the inertial force ${\alpha }_{s}^{*}$ and ${\alpha }_{s}^{*}{t}^{*}$ are changed. Here, ${\mu }_{s}^{*}$ is determined by ${\alpha }_{s}^{*}{t}^{*}$.

Figure 11 shows that a larger $|{\alpha }_{s}^{*}|$, leads to a larger ${\varepsilon }^{*}$ because the apparent inertial force expressed in the second and third terms on the left-hand side of Eq. (21) increases. In addition, Fig. 12 shows that when $|{\alpha }_{s}^{*}|$ is fixed, ${\varepsilon }^{*}$ increases as ${\alpha }_{s}^{*}{t}^{*}$ increases; that is, ${\varepsilon }^{*}$ increases as the dimensionless time ${t}^{*}$ increases. Furthermore, in Fig. 12, it is shown that ${\varepsilon }^{*}$ for ${\alpha }_{s}^{*}<0$ is larger than that for ${\alpha }_{s}^{*}>0$ in this case. It is thought that this occurs because when ${\alpha }_{s}^{*}<0$, the connecting point of bodies A and B approaches the upper end of the pendulum, which is constrained to rotate freely, so that vibrations with high frequency appear at the connecting point and the constraint force increases.

To evaluate the difference ${\varepsilon }^{*}$ caused by considering the internal boundary movement under various numerical analysis conditions, it is necessary to consider the magnitude of the inertial force with respect to the dominant force in the motion. In this flexible pendulum model, the dominant force is dimensionless gravity and the motion of each body is determined by the dimensionless gravity ${g}^{*}= \rho Lg/E$ acting on the entire flexible body. (The other cases are shown in Appendix 3). Therefore, the magnitude of the inertial force with respect to the dominant force is expressed by ${\sigma }^{*}$, which is derived from the following equation.

26 $$ \sigma^{*} = \frac{{\alpha_{s}^{*2} t^{*} }}{{\sqrt {g^{*} } }} = \frac{{\alpha_{s} }}{{\sqrt {Lg} }}\frac{{\alpha_{s} t}}{{L_{s} \left( t \right)}} $$

**Figure 13:**
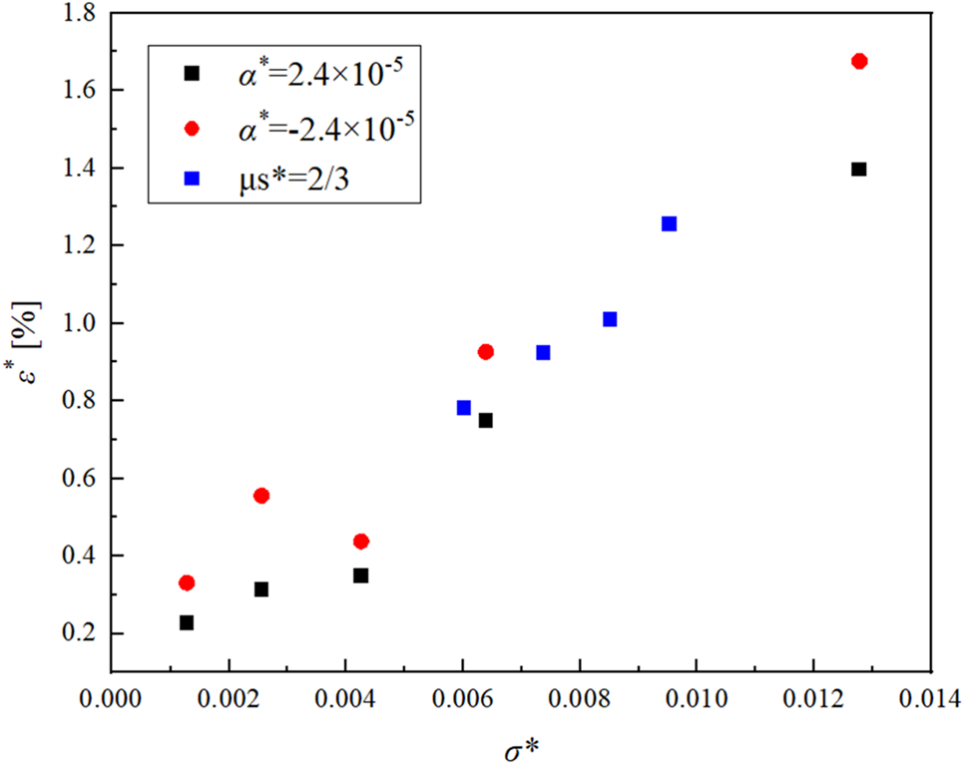
Relationship between ${\sigma }^{*}$ and ${\varepsilon }^{*}$.

Figure 13 indicates that ${\varepsilon }^{*}$ and ${\sigma }^{*}$ have a linear correlation. Therefore, the accuracy of VB-VFE-ANCF is evaluated using ${\sigma }^{*}$. That is, a smaller dimensionless internal boundary movement speed ${\alpha }_{s}^{*}$ and time ${t}^{*}$ and a larger dimensionless gravity ${g}^{*}$ lead to a smaller ${\varepsilon }^{*}$ in nondimension. This corresponds to a smaller internal boundary movement speed ${\alpha }_{s}$ and ${\alpha }_{s}t$ with respect to the total length of the flexible body $L$ and body s length ${L}_{s}(t)$, leading to a smaller ${\varepsilon }^{*}$, in dimension.

From the above, the difference ${\varepsilon }^{*}$ between the numerical results obtained using ANCF and VB-VFE-ANCF can be predicted by ${\sigma }^{*}$. In this case, if ${\sigma }^{*}<0.38$, ${\varepsilon }^{*}$ is within 5%, confirming that the proposed method is useful.

## Conclusion

This study proposed a numerical method named VB-VFE-ANCF for flexible body motion that considers internal boundary movement. This method virtually divides a flexible body into two bodies to avoid the problems associated with internal boundary movement that occur with conventional methods. The analysis show that the accuracy and efficiency of calculations are improved. Because there is no need to recalculate the equation of motion for each element and also approximately calculate that for an element that spans two different environments, as required by conventional methods.

In addition, the proposed method is dimensionless, which further increase the analysis accuracy and efficiency. By using the time-varying length of each body as a representative length, it is possible to convert the target system into a dimensionless system in which the dimensionless length of the body is constant, and perform numerical analysis while maintaining accuracy regardless of the dimensional length change of each body.

Introducing the dimensionless equation of motion, the influence of the inertial force generated by the internal boundary movement, described as a change in the length of each body in the proposed method, was clarified. The validity of this method was shown by comparing its numerical results with those obtained using ANCF, which does not consider internal boundary movement. A function for evaluating the application range of the proposed method was derived.

## Supplementary Information

Supplementary Information.

## Data Availability

The datasets used and/or analyzed during the current study available from the corresponding author on reasonable request.
