# Supplementary material for: Numerical approach for flexible body with internal boundary movement
Source: Sci Rep. 2023 Mar 31;13:5302. doi: 10.1038/s41598-023-32526-3 (PMC10066234; doi:10.1038/s41598-023-32526-3)
Supplement: Supplementary file 1 — Supplementary Information. [file 41598_2023_32526_MOESM1_ESM.docx]

**Appendix 1**

In dimensionless approach, the differential operator is converted as follows;

| $\frac{d}{dt}=\frac{d}{{dt}^{*}}\frac{dt^{*}}{dt}=\frac{1}{T_{R}\mu_{s}^{*}}\frac{d}{{dt}^{*}}$ | (27) |
| --- | --- |

Therefore, the first and second time derivative of nodal coordinates $\mathbf{e}$ are converted to the dimensionless nodal coordinates $\mathbf{e}^{\mathbf{*}}$ as follows;

| $\frac{d}{dt} \mathbf{e}_{i}=\frac{1}{T_{R}\mu_{s}^{*}}\frac{d}{{dt}^{*}}\left( L_{i}\left( t \right)\mathbf{e}_{i}^{*} \right)=\alpha_{i}\mathbf{e}_{i}^{*}+\frac{L_{i}(t)}{T_{R}\mu_{s}^{*}}\dot{\mathbf{e}_{i}^{*}}$ | (28) |
| --- | --- |
| $\frac{d^{2}}{dt^{2}}\mathbf{e}_{i}=\frac{1}{T_{R}\mu_{s}^{*}}\frac{d}{{dt}^{*}}\left( \frac{d}{dt} \mathbf{e}_{i} \right)=2\frac{1}{T_{R}\mu_{s}^{*}}\left( \alpha_{i}-\lambda_{is}^{*}\alpha_{s} \right)\dot{\mathbf{e}_{i}^{*}}+\frac{L_{i}(t)}{T_{R}^{2}\mu_{s}^{*2}}\ddot{\mathbf{e}_{i}^{*}}$ | (29) |

From the above, the equation of motion described in Eq. (21) is derived by substituting Eq. (28) and (29) into Eq. (16) and dividing that by $L_{R}/\left( T_{R}\mu_{s}^{*} \right)^{2}$. Here, $L_{R}/T_{R}^{2}$ is the dimension of the equation of motion and dividing by this means that the equation of motion is made dimensionless using representative length $L_{R}$ and time $T_{R}$.

**Appendix 2**

The difference of the computational time when using ANCF and VB-VFE-ANCF is shown in Figure 14 using a simple flexible pendulum model with an initial angle of 0 degrees, that is vertical state, and its internal boundary moves at constant velocity $\alpha=0.1$ m/s, where $L=10.0$ m for ANCF and $L_{A0}=L_{B0}=5.0$ m for VB-VFE-ANCF and the time step $\Delta t=1.0\times{10}^{-5}$. In this time, when ANCF is used, the boundary position search is performed and the equation of motion is recalculated at each calculation step. Therefore, Figure 14 shows the difference in calculation time depending on whether they are required or not**.** In addition, in order to obtain high accurate results using such ANCF method, the number of elements should be increased since in this ANCF approach, the element that spans two different environments was approximately determine in which environment the element exists. From the above, it is shown that the proposed method reduces calculation cost.


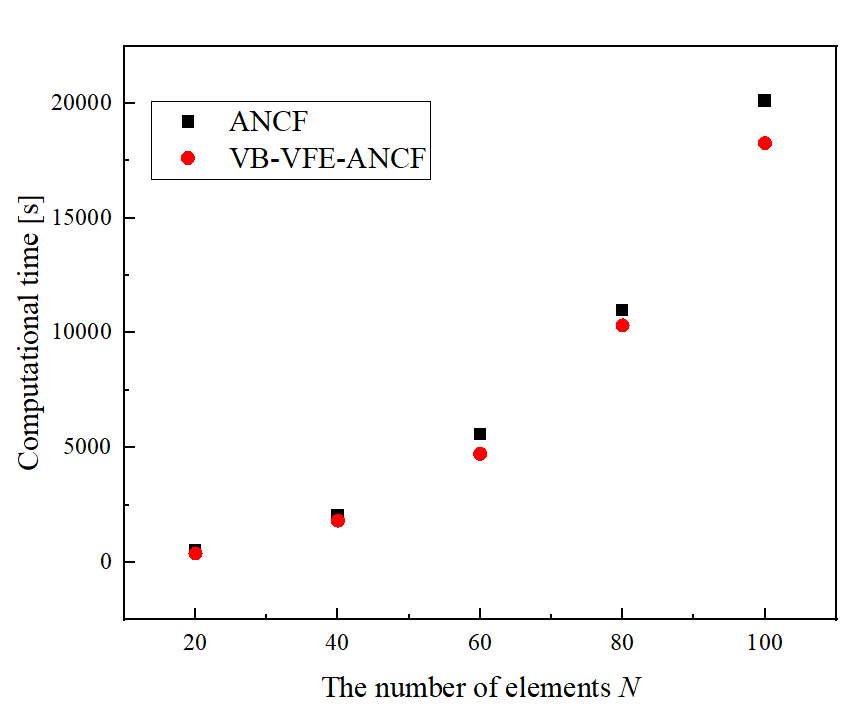

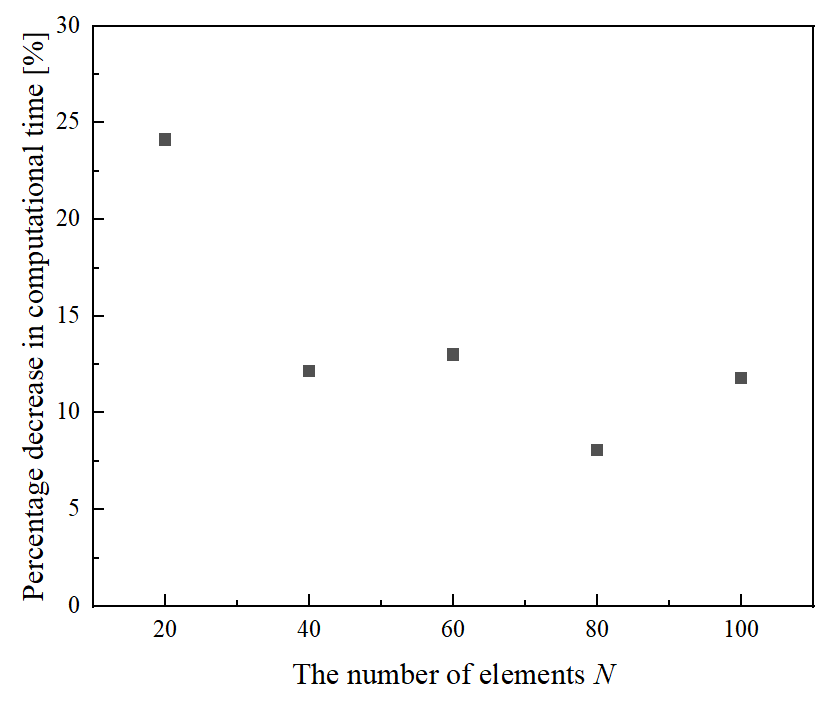


(a) Computational time (b) Percentage decrease in computational time

Figure 14. Comparison of computational time when using ANCF and VB-VFE-ANCF. (a) shows the computational time required to perform numerical analysis with each number of elements, and (b) shows percentage decreases in computational time of VB-VFE-ANCF compared to ANCF.

**Appendix 3**

The indicator $\sigma^{*}$ defined in equation (26) is derived from the balance between gravity and inertial forces because gravity is much larger than other forces in the case of the fluorocarbon pendulum model. Thus, the indicator $\sigma^{*}$, a material-independent indicator, applies if the gravity is much larger than other forces. However, in other cases, another indicator should be considered.

Here, the results obtained using a flexible pendulum model and a flexible beam model of which both the ends are fixed under zero gravity like Figure 10 with 3 materials, (a), (b) and (c) [28-30] are shown in table 1. In both models, $L=1.0$ m for ANCF, $\alpha=0.02$ m/s, $L_{A0}=L_{B0}=0.5$ m for VB-VFE-ANCF, and the time step $\Delta t=1.0\times{10}^{-6}$. In addition, the number of elements is set considering the appropriate value for each material, $N=40$, $N_{A}=N_{B}=20$ for materials (a) and (b), $N=100$, $N_{A}=N_{B}=50$ for material (c).

This table shows that the influence of material parameters is small in the pendulum model and large in the beam model. This is because in the case of a flexible pendulum model, the magnitude of the elastic force varies depending on the material and the error varies slightly, however, the influence of gravity is large compared with them. On the other hand, in the case of a flexible beam model, the error $\varepsilon^{*}$ is depends on $\sqrt{\rho/E}$ included in the coefficient of inertia force since it is under zero gravity and inertia force is larger than the other forces.

Table 1. Relationship between the error $\varepsilon^{*}$ and material properties.

|  | (a) | (b) | (c) |
| --- | --- | --- | --- |
| Material density $\rho$ | $5540$ | $1780$ | $800$ |
| Modulus of elasticity $E$ | $7.0\times{10}^{5}$ | $1.3\times{10}^{9}$ | $1.0\times{10}^{9}$ |
| $\sqrt{\rho/E}$ | $8.9\times{10}^{-2}$ | $1.17\times{10}^{-3}$ | $8.9\times{10}^{-4}$ |
| The maximum error $\varepsilon^{*}$ in 1 second (pendulum) [%] | $0.621$ | $0.268$ | $0.227$ |
| The maximum error $\varepsilon^{*}$ in 1 second (beam) [%] | $6.01\times{10}^{-4}$ | $4.97\times{10}^{-6}$ | $4.64\times{10}^{-6}$ |
